# Latent Transitions of Learning Interests among Kindergarteners in Hakka Bilingual Teaching Programs

**DOI:** 10.3390/children10071273

**Published:** 2023-07-24

**Authors:** Chung-Chin Wu

**Affiliations:** Department of Early Childhood Education, National Pingtung University, Pingtung 900391, Taiwan; minin72704@mail.nptu.edu.tw

**Keywords:** bilingual program, Hakka, kindergartener, latent transition, learning interest

## Abstract

The profiles of kindergarteners’ learning interests in Hakka bilingual teaching programs are unclear and the effects of these programs on the transition of such interests over the long term are under investigation. This study analyzed the learning interest profiles of 112 kindergarteners (data gathered by kindergarten teachers) enrolled in immersion/non-immersion Hakka bilingual programs in Taiwan. Latent transitions in these profiles were analyzed based on pre- and post-implementation data. The results showed that two different subgroups were identified based on the kindergarteners’ learning interest profiles before and after the implementation of the Hakka bilingual program. The pre-implementation subgroups contained the “moderate situational and low-to-moderate individual interest” and the “high situational and moderate-to-high individual interest” profiles. Post-implementation subgroups consisting of “moderate-to-high situational and moderate individual interest” and “high situational and individual interest” profiles were identified. Moreover, four patterns of transition in the kindergarteners’ learning interest profiles were uncovered: (1) a slight increase in both learning interests, (2) a significant increase in both learning interests, (3) a slight regression in both, and (4) a maintenance of situational interest coupled with a slight increase in individual interest. Lastly, the non-immersion Hakka program showed significant and more positive effects on the “moderate situational and low-to-moderate individual interest” profile subgroup compared to the equivalent group from the Hakka immersion program. These results provide new evidence complementing previous findings reached via different analytical approaches and contribute to the overall conclusion that bilingual programs improve learning outcomes.

## 1. Introduction

Second language (L2) teaching is challenging—particularly with young learners—and is dependent on students’ interest in learning [1], which also positively impacts their short- and long-term academic achievement [2,3]. Therefore, the promotion of interest in L2 learning should precede attempts to teach the L2 itself. Indeed, this approach is typical of most interventions developed to benefit young children’s L2 learning. However, research is yet to adequately address how students’ learning interest changes according to the particular L2 learning environment.

Hakka is officially recognized as one of Taiwan’s endangered languages, with the country’s government investing over 100 million dollars annually over the past two decades to promote its usage [4]. Bilingual programs at various levels now teach Hakka as an L2 in addition to Chinese; over the past 15 to 20 years, Taiwanese kindergartens have offered Hakka immersion and non-immersion bilingual teaching programs to enable students to learn the language as early as possible [5,6]. However, the literature review located only a few researchers focused on probing kindergarten [7] or elementary school teachers’ viewpoint of the Hakka language program [8,9]. No research directly investigating the effectiveness of either program in building kindergarteners’ interest in learning Hakka exists, a gap that the present study aimed to fill.

Learning interest is a multi-dimensional construct composed of situational and individual interests. *Situational interest* is a short-term state that can be easily elicited or eliminated by the presence or absence of situational characteristics (e.g., interesting teaching and learning activities). In contrast, *individual interest* is a long-lasting trait that develops through a long-term positive affect or value attached to specific learning material or content [10]. Recent research evidence supports the validity of this two-factor framework for understanding the L2 learning interest of kindergarteners [11]. However, many previous studies have either treated learning interest as a unidimensional construct or focused on one or another of its aspects [12,13,14]. Among these studies, only a few have addressed long-term changes in learning interest or its effect on learning outcomes (e.g., engagement levels in class) in school settings. For example, Vongkulluksn et al.’s semester-long study of middle- and upper-elementary school students demonstrated a general decline in situational interest that contrasted with the high levels maintained by a design-based makerspace course [15]. Alexander et al. found that science interest (represented by preferences in choosing science activities) in preschool students positively impacted engagement in informal science activities [16]. Preschoolers’ interest in science learning was also positively associated with stronger self-concept and achievement in science among elementary students [17].

Despite their robustness, the above studies contain several limitations. First, they segment individual learning interest into either its situational or individual forms using general linear models such as structural equation modeling. Yet, such approaches do not explain how people might simultaneously express different levels of situational vs. individual interest as shared learning interest profiles. Second, they do not evaluate how certain L2 teaching programs contribute to changes in students’ learning interests over time. Consequently, they cannot reliably inform teachers’ efforts to plan timely and adaptive interventions for students whose learning interests differ. However, the application of latent profile analysis enables individuals with similar learning interests to be classified into homogenous subgroups while also identifying heterogeneity between these subgroups. Sequentially, latent transition analysis can be used to investigate how certain teaching programs contribute to changes in students’ learning interests by tracing movements from one subgroup with a particular learning interest profile at the start of a semester to another with a different profile at the semester’s end.

Research has highlighted the positive impacts of bilingual immersion programs on general academic performance as well as L2 learning performance across multiple languages [18,19,20,21,22,23,24,25,26,27,28,29,30]. However, no direct evidence exists to confirm the positive effect of bilingual immersion programs on learning interest. One indirect exception was the finding that early participation in an immersion French program produced more positive attitudes toward French Canadian culture compared to the equivalent English program [31,32]. However, the result is not directly relevant to the effects of Hakka bilingual programs on learning interest, and it therefore remains unclear whether and to what extent the Hakka bilingual programs contribute to transitions in learning interest profiles among individuals in different subgroups.

To fill the current gaps in knowledge indicated above, the main purposes of this study are as follows:

- To investigate whether different learning interest profiles form among kindergarteners over time.
- To clarify whether and to what extent two specific Hakka bilingual programs contribute to transitions in learning interest among individuals in different subgroups.

## 2. Methodology

### 2.1. Participants and Setting

A total of 112 kindergarteners aged 5 years (60 males and 52 females) in four classes were selected from Taiwanese preschools. Two of these preschools ran a Hakka immersion program (Hakka as the primary language; Chinese the secondary language) and two ran a Hakka non-immersion program (Chinese as the primary language; Hakka the secondary language). An equal number of kindergarteners (56) was selected from each program type. The stated aim of both programs was to preserve the Hakka language for future generations. The Hakka immersion programs were defined after Genesee [33] as programs where students spend at least 50% of their time using Hakka and the rest of the time using Chinese in their daily schooling, with the reverse holding for the non-immersion programs.

The programs were based on the most widespread instructional approach in Taiwanese preschools, which integrates several related teaching and learning activities into a single theme. In the Hakka immersion program, teachers use Hakka for classroom activities, supplementing this with Chinese to boost understanding of difficult concepts. Typically, some Hakka songs and rhythms are incorporated into classroom activities. In the non-immersion Hakka program, teaching and learning activities are similar but larger proportions of Chinese are used. The second main difference between the non-immersion and immersion programs is that the latter includes more outdoor visiting activities to bring kindergarteners into Hakka communities to experience the culture and interact with local residents [5]. These Hakka language programs were implemented with a duration of three months. The Hakka immersion program was implemented in every daily teaching activity, while the Hakka non-immersion program was implemented in a forty-minute lesson taught by professional Hakka teachers once a week with a total of twelve lessons, and kindergarten teachers sometimes used the Hakka language to replace the Chinese in their daily teaching activities.

### 2.2. Measures

This study adopted a scale originally developed to measure and understand kindergarten boys’ and girls’ learning interest in a Hakka bilingual program [11]. It consisted of two sub-scales measuring situational and individual interest, each with four items. A sample item for situational interest was “Jason is fascinated by the instructional method and teaching materials in the Hakka bilingual program,” and for individual interest was “Jason is looking forward to engaging in Hakka teaching and learning activities” (Chinese pseudonyms were used to replace the names in the items). The kindergarten teachers then rated each kindergartener for their similarities to the item descriptions on a 6-point Likert scale (1 = complete mismatch; 6 = complete match) based on their observations from the Hakka language program. The same learning interest scale for kindergarteners was implemented at the beginning of the semester and again at the end, following a three-month interval. The reliabilities of individual items in the situational interest sub-scale measured at the start and the end of the semester were 0.95 and 0.93, respectively. The reliabilities of individual items in the individual interest sub-scale measured at the start and the end of the semester were 0.96 and 0.97, respectively.

### 2.3. Analysis

The latent transition analysis (LTA) undertaken to analyze the latent transition of learning interest profiles in the Hakka bilingual programs consisted of five steps [34,35].

Step 1: Estimating an unconditional latent program analysis (LPA) model for each separate time point.

This step aimed to determine the number of latent learning interest profiles at each time point. Each profile contained members with similar learning interests at quantitatively different levels. Several LPA models with increasing numbers of latent profiles were analyzed using criteria AIC (Akaike information criterion), BIC (Bayesian information criterion), and ABIC (sample-size-adjusted BIC), and the log-likelihood ratio tests LMR LRT (Lo–Mendell–Rubin adjusted likelihood ratio test) and BLRT (bootstrapped likelihood ratio test) were used to determine the optimal LPA model at each time point for subsequent analysis. The model with *k* latent profiles (containing the lowest values of AIC, BIC, ABIC, and non-significant LRT test results) was compared to the model with k+1 latent profiles and retained for follow-up analysis.

In addition to the above information criteria and LRT tests, entropy was also used to evaluate the models’ classificatory quality, with a threshold value of 0.80 [36,37]. An additional criterion was the number of memberships in each latent profile, with those containing less than 5% of the sample considered meaningless [38].

Step 2: Determining the best LTA model.

After identifying the optimal LPA models at two time points, the corresponding latent profiles were incorporated into a single LTA model to be analyzed. The above information criteria, LRT tests, and standards were reapplied at this step to obtain the optimum LTA model for later analysis.

Step 3: Testing longitudinal measurement invariance in multi-group LTA.

The longitudinal measurement invariance of multi-group LTAs was then analyzed based on the optimal model determined in step 2. Two LTA models, measurement invariance (M_0_) and measurement non-invariance (M_1_), were analyzed. The item-response probabilities at each time point were set at equal levels in the former model but were freely estimated in the latter. While AIC, BIC, and ABIC were also applied at this step, the LR test was replaced by the MLR (maximum likelihood with robust standard errors) estimator. The following formula was applied to test the differences between M_0_ and M_1_ [37]:

$$\mathrm{TRd}=\frac{-2\times \left(L_{0}–L_{1}\right)}{\mathrm{cd}};\;\mathrm{cd}=(p_{0}\;\times \;c_{0}\;-p_{1}\;\times \;c_{1})/(p_{0}\;-\;p_{1})$$

where L_0_ and L_1_ were the log-likelihood values of the measurement invariance (M_0_) and measurement non-invariance models (M_1_), respectively, p_0_ and c_0_ were the number of free parameters and scaling correction factors of M_0_, respectively, and p_1_ and c_1_ represented the number of free parameters and scaling correction factors of M_1_, respectively. The significant TRd result indicated that the LTA model was not measurement invariant.

Step 4: Defining the latent profiles.

After identifying the best multi-group LTA model, the latent profiles were defined and labeled according to the quantified patterns of learning interest they contained. The kindergarteners in each profile expressed similar quantified patterns of learning interest.

Step 5: Estimating the latent profile prevalence and transition probabilities for the two programs.

*Latent profile prevalence* describes the percentages of kindergarteners within each latent profile, while *transition probability* expresses the likelihood that the kindergarteners’ learning interest would shift from one latent profile at time 1 to another at time 2 (i.e., pre- and post-implementation of the Hakka bilingual programs).

## 3. Results

### 3.1. The Latent Profiles of Learning Interest at Two Time Points

Table 1 shows the LPA of learning interest before and after the Hakka bilingual programs. At time 1, the model with five latent profiles displayed the lowest AIC, BIC, and ABIC values (1830.27, 1917.63, and 1807.29, respectively). However, the LMR LRT indicated that this model did not differ significantly from the model with four latent profiles (LMR LRT *p* > 0.05), which in turn resembled the model with three profiles (LMR LRT *p* > 0.05). This latter model was chosen for subsequent analysis since it was significantly better than the two-profile model (LMR LRT *p* = 0.03 > 0.05) and had the highest entropy value (0.96). However, the model contained only 22 kindergarteners in one of the three latent profiles, suggesting it might have insufficient kindergarteners in its subgroups for subsequent analysis. In addition, the entropy of this model was approximately equal to that of the model with two latent profiles. This raised the possibility that the model with two latent profiles at time 1 might have been a better candidate for later analysis.

At time 2, similarly, the model with five latent profiles displayed the lowest AIC, BIC, and ABIC values (1516.86, 1658.22, and 1493.88, respectively) among the learning interest models. Again, the LMR LRT revealed significant differences between this model and the one with four latent profiles (LMR LRT *p* > 0.05). While the model with five latent profiles appeared optimal, one of its profiles contained only one kindergartener and its entropy value was below the four-profile model, meaning it could not be considered optimal. Among the following four options, the model with a single latent profile performed best on the LR tests suggested but its AIC, BIC, and ABIC values were significantly higher than the alternatives, also ruling it out. Among the remaining models, the four-profile model had the lowest AIC, BIC, and ABIC values and the highest entropy—but one of its latent profiles contained only one kindergartener. Similarly, the AIC, BIC, and ABIC values of the model with three latent profiles were lower than that of the model with two latent profiles; this model also had higher entropy but fewer kindergarteners (12) in one of its three latent profiles, while at least 36 kindergarteners occupied one of the latter model’s two profiles. Thus, at time 2, the two-profile model appeared the best candidate for subsequent analysis. However, because the number of kindergarteners in latent profiles might increase due to the greater statistical power of LTA [34], both the two- and three-profile models were temporarily included at both time points for later analyses.

Four LTA models (2–2, 2–3, 3–2, and 3–3) were then analyzed, with the former and latter numbers representing the number of latent profiles at times 1 and 2, respectively. As Table 2 shows, the 3-3 LTA model included the lowest AIC, BIC, and ABIC values (3793.68, 3989.42, and 3761.87, respectively) and the second highest entropy value of 0.97. In contrast, the 2–2 LTA model had the highest AIC, BIC, and ABIC values (4288.50, 4427.14, and 4265.96, respectively). However, the latter three LTA models had at least one latent profile containing fewer than 5% of kindergarteners. In contrast, the latent profiles in the 2–2 LTA model all exceeded 30% of the sample, and its entropy was comparable to the other three LTA models, which demonstrated excellent classification quality. Overall, the 2–2 LTA model was determined to be the only model that might produce meaningful results in the subsequent analysis.

Two models with (M_0_) and without (M_1_) longitudinal measurement invariances were analyzed based on the determined LTA model, excluding the grouping variable. The M_0_ had higher AIC, BIC, and ABIC values (4526.02, 4626.60, and 4509.67, respectively) than the M_1_ (4443.95, 4588.03, and 4420.53). The log-likelihoods for M_0_ and M_1_ were −2226.01 and −2168.97, while the free parameters recorded for each were 37 and 53 and the scaling correction factors were 2.5825 and 1.7852, respectively. The TRd result was also significant, 121.76 under a df of 16 with *α* set at 0.05, suggesting that the item-response probabilities of learning interest measurements at the two time points were non-invariant and should allow separate estimates across time.

### 3.2. The Latent Transition Analysis of Learning Interest in Immersive and Non-Immersive Hakka Bilingual Programs

The latent profiles at two time points were defined before estimating their prevalence and the transition probabilities for each language program. Table 3 shows the mean scores for each of the learning interest items used to define the latent profiles at times 1 and 2 in the LTA model alongside the numbers and proportions of kindergarteners in each profile. Learning interest was categorized as “low”, (1–2) “low-to-moderate”, (2–3) “moderate”, (3–4) “moderate-to-high”, (4–5), and “high” (5–6). At time 1, the first latent profile contained “moderate situational interest” (mean values of 3–4) and “low-to-moderate individual interest” (*M* = 2–3). The second latent profile at time 1 was characterized by “high situational interest” (*M* = 5–6) and “moderate-to-high individual interest” (*M* = 4–5). At time 2, the first latent profile was marked by “moderate-to-high situational interest” (*M* = 4–5) and “moderate individual interest” (*M* = 3–4), while the second consisted of “high individual and situational interest” (*M* = 5–6 for both).

For 3-year-old preschoolers, the AIC, BIC, and ABIC values of the model with eight latent profiles were the lowest. However, the likelihood ratio test for the differences between the models with eight and the seven latent profiles did not reach significance (LRT and BLRT *p* values were 0.40 and 1, respectively). It seems that the model with seven latent profiles should be the best, but the models with two, three, and four latent profiles have entropies greater than 0.98, which is much higher than 0.89 for the model with seven latent profiles. Both the models with three and four latent profiles have few preschoolers in one of those subgroups (the proportions are just 2.97 and 0.95%, respectively). In contrast, the proportions of the subgroups in the model with two latent profiles were greater than 20%. However, it seems that the subgroups were too few in this model to better explain the motor development for preschoolers, and the statistics power may be increased to classify more preschoolers into subgroups in the latent transition analysis. Consequently, the model with three latent profiles was temporarily chosen as a basis for later analysis.

Table 4 displays the latent transition probabilities of learning interest for the non-immersive and immersive Hakka programs from time 1 to time 2. First, in the non-immersive Hakka program, 30 out of all 56 kindergarteners comprised the “moderate” (situational interest) and “low-to-moderate” (individual interest) latent profile at time 1. By time 2, the learning interests of these 30 students had shifted: 17 (58%) now matched the “moderate-to-high and moderate” profile while 13 (42%) fitted the “high situational and individual interest” profile. The remaining group of 26 students (43.46%) matched the “high and moderate-to-high” latent profile at time 1. By time 2, the learning interests of 5 of these children (18%) had slightly declined, whereas 21 (82%) members of the sample had retained their situational interest at the same “high” level, with their individual interest increasing from a “moderate-to-high” to a “high” level.

Among the 56 kindergarteners enrolled in the immersive Hakka program, 10 belonged to the “moderate and low-to-moderate” latent profile at time 1. By time 2, each of these students’ levels of situational and individual learning interest had increased to “moderate-to-high and moderate” levels, respectively; no learner had transitioned to a “high/high” latent profile. The other 46 children (82.14%) fitted the “high and moderate-to-high” latent profile at time 1. By time 2, 8 students’ (13%) level of learning interests had slightly declined, while 38 (87%) had retained their situational interest at the same “high” level and slightly increased their individual interest from a “moderate-to-high” to a “high” level.

## 4. Discussion and Conclusions

Overall, the results revealed shifts in the children’s learning interest profiles over time. Before the Hakka teaching program was implemented, the two subgroup profiles were labeled “moderate situational interest; low-to-moderate individual interest” and “high situational interest; moderate-to-high individual interest”. Post-implementation, these subgroups were characterized by the “moderate-to-high situational interest; moderate individual interest” and “high situational interest; high individual interest” profiles. Four transition tendencies were identified: (1) a slight increase in both learning interests, (2) a significant increase in both learning interests, (3) a slight regression in both learning interests, and (4) a maintenance of situational interest coupled with a slight increase in individual interest.

Pre-implementation, the “moderate situational interest and low-to-moderate individual interest” latent profile in the Hakka non-immersion program was about three times more prevalent (53.57%) than in the Hakka immersion program. After a three-month interval, the non-immersion students had either transited to the “moderate-to-high and moderate individual interest” subgroup (about 60%) or the “high in both interests” subgroup (about 40%). However, in the immersion program, relatively few kindergarteners (10; 17.86%) had shifted to the “moderate-to-high and moderate individual interest” subgroup.

Before implementation, the latent profile “high situational interest and moderate-to-high individual interest” in the Hakka immersion program was about two times larger (82.14%) than in the non-immersion equivalent. When the program ended after three months, an equal proportion of this group of kindergarteners in both this and the non-immersion program had shifted to the “moderate-to-high and moderate individual interest” (18%) and “high in both learning interests” (82%) subgroups. Taken together, these results suggest that the Hakka non-immersion program more strongly boosts all levels of both learning interests and is equally effective in preventing their decline compared with the immersion program. In contrast, the Hakka immersion program was only slightly effective in increasing “low-to-moderate” learning interests and maintaining situational interests/enhancing individual interests among kindergarteners with “moderate-to-high” learning interest profiles.

Overall, the findings of this study provide new evidence for the learning profiles of kindergarten students. They also complement previous research demonstrating the positive effects of L2 immersion programs on learning/achievement performances [18,19,20,21,22,23,24,25,26,27,28,29,30] and attitude [31,32]. Surprisingly, the Hakka non-immersion program appeared to promote the learning interests of all kindergarteners—regardless of the level of these interests—more effectively than the immersion program. This study therefore provides a timely reminder of the advantages of Hakka non-immersion programs, whose linkage of the language to students’ daily life would help them understand local Hakka communities and culture, boosting their learning interest. It also contributes to learning interest theory by highlighting elements linked to children’s individual and situational interests.

Despite the above significances or contributions of this study, there were also some limitations. First, the kindergarteners in this study were not a nationally representative sample, which may limit the generality of the present findings. Second, this study did not intervene in the instructional practices of both Hakka language programs to make kindergarteners’ learning interests in these courses and activities in these programs comparable, which means that the incongruencies of teaching and learning content and activities among these two programs may be possible confounding causes of the differences in learning interests. This suggests that standardized course design and materials for both program types were needed to expend the internal validity of the results in this study. Future studies are encouraged to recruit a nationally representative sample and to adopt a standardized teaching course and standardized teaching materials to re-examine the present findings. Specifically, future studies can adopt stratified random sampling to assure the recruiting of more nationally representative kindergarteners in the implemented Hakka language program. In addition, it is encouraged to adopt the same theme and corresponding teaching and learning content and activities, and to fix the duration of each lesson or activity as much as possible; however, the main difference, the proportion of the uses of Hakka–Chinese between these programs, should be left constant. Consequently, the results would be more comparable, and corresponding findings would be expected to be more convincing.

## Figures and Tables

**Table 1:** Results of LPA at two time points.

Models	Log-Likelihood	AIC	BIC	ABIC	LMR LRT *p*	BLRT *p*	Entropy
Time 1							
1 profile	−1360.56	2755.11	2798.61	2748.04	–	–	–
2 profiles	−1087.76	2225.53	2293.49	2214.48	0.046	0.00	0.95
3 profiles	−961.49	1990.99	2083.42	1975.97	0.03	0.00	0.96
4 profiles	−899.46	1884.92	2001.82	1865.93	0.37	0.00	0.95
5 profiles	−863.13	1830.27	1971.63	1807.29	0.89	0.00	0.94
Time 2							
1 profile	−1288.10	2608.21	2651.70	2601.14	–	–	–
2 profiles	−1016.22	2082.44	2150.41	2071.40	0.35	0.00	0.95
3 profiles	−885.97	1839.94	1932.37	1824.92	0.38	0.00	0.97
4 profiles	−738.98	1563.95	1680.85	1544.95	0.62	0.00	1.00
5 profiles	−706.43	1516.86	1658.22	1493.88	0.17	0.00	0.96

**Table 2:** Results for LTA models excluding the grouping variable.

Model	AIC	BIC	ABIC	Entropy
2–2 transition model	4288.50	4427.14	4265.96	0.95
2–3 transition model	4043.39	4209.22	4016.44	0.97
3–2 transition model	4050.65	4216.48	4023.70	0.95
3–3 transition model	3793.68	3989.42	3761.87	0.97

Note: The numbers to the left and right of the hyphen denote the number of latent profiles at the first and second time points, respectively.

**Table 3:** Mean scores for each learning interest item and the numbers and proportions of kindergarteners in each latent profile in the LTA model.

Dimension and Items	Latent Profiles for Time 1 (Number/Proportion of Kindergarteners)		Latent Profiles for Time 2 (Number/Proportion of Kindergarteners)	
	1 (30/53.57%)	2 (26/46.46%)	1 (24/42.86%)	2 (32/57.14%)
Situational interest				
Item 1	3.87	5.34	4.69	5.80
Item 2	3.56	5.19	4.25	5.68
Item 3	3.87	5.30	4.45	5.71
Item 4	3.94	5.34	4.50	5.78
Individual interest				
Item 1	2.97	4.81	3.67	5.41
Item 2	2.99	5.06	3.60	5.65
Item 3	2.69	4.38	3.25	5.05
Item 4	2.36	4.42	3.20	5.11

**Table 4:** Transition probabilities in the latent transition models of two Hakka bilingual teaching programs.

Latent Profile at *t*_1_ (Number/Proportion of Kindergarteners)	Hakka Non-Immersion Teaching Program	
	Latent Profile at *t*_2_ (Number of Kindergarteners)	
	“Moderate-to-High; Moderate”	“High; High”
“Moderate; low-to-moderate” (30/53.57%)	0.58 (17)	0.42 (13)
“High; moderate-to-high” (26/43.46%)	0.18 (5)	0.82 (21)
	Hakka immersion teaching program	
	“Moderate-to-high; moderate”	“High; high”
“Moderate; low-to-moderate” (10/17.86%)	1.00 (10)	0.00 (0)
“High; moderate-to-high” (46/82.14%)	0.18 (8)	0.82 (38)

Note: The strength to the left and right of the comma denote the strength of situational and individual interest, respectively.

## Data Availability

The data presented in this study are available on request from the corresponding author. The data are not publicly available due to research ethics statements which were declared in the informed consents.
